# Development and validation of a pre-scoring system for nonspecific low back pain among general population in Guangzhou: a cross-sectional study

**DOI:** 10.1186/s12889-019-7564-9

**Published:** 2019-09-12

**Authors:** Kai Wang, Jing-wen Zhang, Shao-xiong Min, Xin-yi Xu, Sheng-li An

**Affiliations:** 1 Department of Biostatistics, School of Public Health (Guangdong Provincial Key Laboratory of Tropical Disease Research), Southern Medical University, No. 1838 North Guangzhou Avenue, Guangzhou, 510515 People’s Republic of China; 2 Department of Epidemiology and Biostatistics, School of Public Health, Tongji Medical College, Huazhong University of Science and Technology, No. 13 Hangkong Road, Wuhan, 430030 People’s Republic of China; 3 Orthopaedic Center, Zhujiang Hospital, Southern Medical University, No. 253 Gongye Avenue, Guangzhou, 510282 People’s Republic of China

**Keywords:** Non-specific low back pain, Associated factors, Pre-scoring system, General population

## Abstract

**Background:**

Nonspecific Low Back Pain (NLBP) is a common disease with a low cure rate and significant impact on the population. This study aimed to develop and validate a pre-scoring system for identifying the risk of suffering from NLBP among the general population in Guangzhou.

**Methods:**

A total of 1439 eligible subjects were surveyed in Guangzhou by stratified random sampling and was divided randomly into the development dataset (69.6%) and validation dataset (30.4%) subsequently. Based on the development dataset, potential associated factors (average exercise times weekly, the intensity of daily work, etc.) with NLBP were tested by the sequential logistic regression, and a pre-scoring system was formulated with Sullivan’s method and graded afterward. The internal validity of the system was assessed by AUC and calibration plot, and the external validation was performed in the validation dataset.

**Results:**

The prevalence rates of NLBP in the development dataset and the validation dataset were 12.97 and 13.27%, respectively. Age, BMI, average exercise times weekly, gender, educational level, the intensity of daily work, place of residence, monthly income, overall evaluation of health condition and physiology health were identified as significant factors. The total risk score ranged from 0 to 38, which was split into three risk grades: low risk (0 to 18), intermediate risk (19 to 22) and high risk (23 to 38). The pre-scoring system had an adequate calibration and a good discriminating ability with bootstrap-corrected AUC equaling 0.861 in the development dataset and 0.821 in the validation dataset.

**Conclusions:**

A pre-scoring system that could help clinicians to assess the risk of NLBP in the general population was validated. Further validation of the system in a new population or prospective cohort study is suggested.

**Electronic supplementary material:**

The online version of this article (10.1186/s12889-019-7564-9) contains supplementary material, which is available to authorized users.

## Introduction

Non-specific low back pain (NLBP) is defined as tension, soreness and/or stiffness that exists in the lower back region for which there is not a specific cause of the pain [1–6]. As a severe public health problem in the world for many years, NLBP has an approximate demission rate of 39% from work and meanwhile it has been one of the most common reasons for using complementary and alternative medicine [7–10]. The prevalence of NLBP ranges from 10 to 49% among the population of different ages, and even as much as 60–85% during an individual’s lifetime [11–13].

Literature indicates NLBP attributes to multiple risk factors, which include gender [12], smoking [14], BMI [7] and improper sitting posture [15], etc. More studies show that females have a higher prevalence of LBP across all age groups than males, and postmenopausal women are more susceptible to it than young or middle-aged women due to female hormone fluctuation and menstruation [12]. Given lifestyle, sedentariness or long-standing for over 2 h has been found to increase the likelihood of having NLBP [15]. Clinically, such problems as scoliosis, low back muscle endurance, abnormal trunk mobility, and muscle imbalance are higher risk factors [16–20].

VonKorff and Miglioretti [21] developed a risk score system to identify patients at risk of chronic LBP, which included pain severity degree, interference with usual activities number of other pain and number of days with back pain in the prior 6 months. Hill et al. [22] developed a brief screening tool to identify subgroups of patients for initial treatment in primary care. Janwantanakul et al. [23] built another risk scoring system to identify office workers susceptible to LBP based on a prospective cohort study. However, their findings, along with other literature, are somewhat inconsistent and not validated by external data [23–28]. To our knowledge, no scoring system to identify the general population at risk of NLBP has been comprehensively established. The purpose of the present study was to construct a pre-scoring system to assist health care providers in identifying individuals’ potential probability of suffering from NLBP with only several readily available clinical data.

## Material and methods

### Source of data

This field investigation was conducted from August 2013 to May 2014, and a total of 2100 participants were surveyed using a stratified sampling approach. Briefly, in stage 1, as the Nansha district is newly established and geographically far from the city centre of Guangzhou, we selected the remaining 11 districts in Guangzhou. One community from each district was randomly selected in stage 2. Finally, we randomly selected individuals from the selected communities (age 20–59). And only one participant was selected from every household. The work was approved by the Institutional Review Board at Zhujiang Hospital, Southern Medical University, Guangzhou, China (NO. 2013-BLK-009).

### Outcome definition

The body diagram from the standardised Nordic questionnaire was used to identify the location of low back pain [29]. In the questionnaire, the following questions were for the primary diagnoses of NLBP, “Have you ever been diagnosed with such lumbar diseases as a lumbar disc herniation, lumbar hyperosteogeny, lumbar muscle strain, lumbar degenerative disease or rheumatism?” “Is this the first time for you to suffer from low back pain?” “How long does your low back pain last?” In this study, low back pain lasted for a duration between 6 weeks and 12 months, and confirmed not from lumbar diseases was defined as NLBP [1].

### Participants and predictors

A questionnaire was designed for the survey, in which participants’ private information was omitted. All participants were informed consent and voluntary in this survey.

The inclusion criteria were as follows: (1) age between 20 and 59 years, (2) no deformity or asymmetry in the spine or lower limbs, (3) no mutilation, (4) no problems in reading and communication [23].

The exclusion criteria were as follows: (1) ischialgia resulting from lumbar spinal stenosis, a tumour of the spine, etc., (2) reported pregnancy or spinal, intra-abdominal or femoral surgery in the past year and (3) musculoskeletal, rheumatic, orthopaedic, somatic or psychiatric disorders [7, 12, 30].

The researchers were trained in advance to assist the participants in completing the questionnaire, which included demographic, work-related and psychosocial data as well as the presence of NLBP.

The demographic data included birthday, height, weight, gender, nation, educational level, smoking habits, drinking habits, marital status, average exercise times weekly, place of residence and monthly income.

The work-related factors included the type of occupation, main nature of work, the intensity of daily work, job position and exposure or not to any vibration sources at work.

To assess the quality of life, the participants were also asked to complete the Chinese abbreviated version of World Health Organization Quality of Life (WHO-QOL-BREF-Chinese)-Brief, which consisted of 26 items in four domains (physiological health, psychological health, social relation health and environmental health) and two general evaluations about the quality of life and health condition.

Pre-survey was conducted three times to correct items and evaluate the reliability and validity of the questionnaire before formal data collection. The content validity of the questionnaire was assessed by six experienced reviewers, including one biostatistician, one epidemiologist, two surgeons, one physician, and one community manager. The Cronbach’α was 0.818, which displayed an acceptable outcome.

### Sample size and missing data

It was difficult to calculate the sample size for the observational study, especially in multivariable regression model settings. We used the rule of thumb recommended by Peduzzi et al. [31] and Harrell et al. [32], namely, events per variable (EPV) being 10 or higher in our study. If there were about ten significant associated factors with NLBP, a minimum of 100 (10 × 10) participants should have the event in the sample.

Since the data had no more than 2% missing values, we imputed it with the EM algorithm to assure the stability of the results. All results were based on the imputed complete dataset.

### Statistical analysis

Continuous variables were expressed as Mean ± S.D., while categorical or ordinal variables were expressed as absolute (n) and relative (%) frequency. All the subjects were randomly divided into two sets, a development (69.6%) dataset, and a validation (30.4%) dataset. Three steps were taken to develop the pre-scoring system based on the development dataset. Firstly, we conducted univariate logistic regression to select possible associated factors with a *P*-value ≤0.1. Then, a backward logistic regression was used to select potential associated factors (demographic and work-related factor) and to construct a basic model. Finally, psychosocial factors, four domains and two general evaluations about quality of life, were evaluated sequentially based on the above basic model. And a risk model was developed sbsequently. The incremental prognostic usefulness of psychosocial factors was evaluated by the integrated discrimination improvement (IDI) and the continuous net reclassification improvement (NRI) [33].

Based on the developed risk model, we created a simple pre-scoring system subsequently by Sullivan et al.’s method [34]. Firstly, we classified the continuous variables into categories in terms of clinical significance. Secondly, we specified the mid-point value as the reference value for each category. To determine the reference values for the first and last categories of continuous variables, we use the 1st and the 99th percentile to minimize the influence of extreme values. Thirdly, the lowest risk category of each variable was served as the base category. The difference of reference values between each category and base category multiplied by the regression coefficient of the corresponding variable in the risk model was defined as the distance of each category from the base category. Fourthly, One score of the scoring system was defined as a constant of 0.48 which means the increase of risk associated with a 5-year increase in age (0. 096 × 5). Finally, the base category of each variable was assigned 0 scores. And the score of other categories was computed by dividing corresponding distance with the constant of 0.48 and then rounded to the nearest integer.

The score was then summed to create a total risk score for each participant, and the participants were classified into three grades: low risk, intermediate risk, and high risk of NLBP.

The discrimination of the models and the system were measured by Areas Under the ROC Curve (AUC). Calibration of predictions was assessed by the calibration plot. The internal validity of the system was assessed by bootstrap techniques, and the external validation was performed in the validation dataset.

If the correlation coefficient between variables was ≥0.60, only the variable judged to be more clinically relevant was included in the model. Confirmatory factor analysis was conducted to recheck the structure validity of the WHO-QOL-BREF-Chinese. All statistical calculations were performed on SAS software (v. 9.3; SAS Institute Inc., Cary, NC). A 2-tailed *P* value < 0.05 was considered as statistically significant.

The reporting of the present study closely follows the Transparent Reporting of a multivariable prediction model for Individual Prognosis Or Diagnosis (TRIPOD) statement [35].

## Results

### Characteristics of the participants

**Fig. 1:**
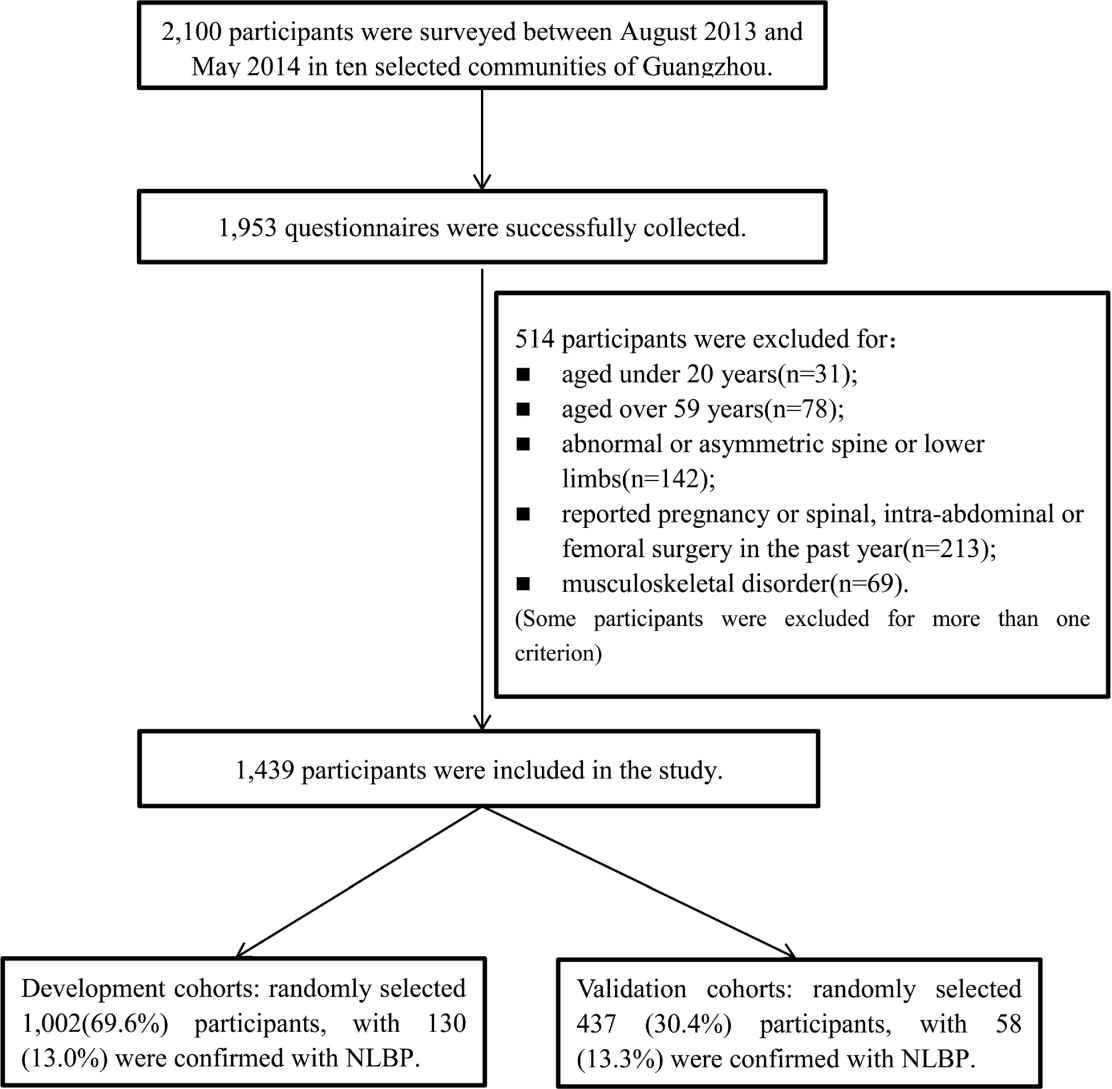
Flow diagram of enrolling the participants at each stage of study

The flow chart of participants is presented in Fig. 1. Totally, 2100 questionnaires were distributed and a total of 1953 ones were successfully collected, with a response rate of 93.0%. Among them, 514 were excluded, including 31 aged under 20 years, 78 aged over 59 years, 142 with abnormal or asymmetric spine or lower limbs, 213 reported pregnancies or spinal, intra-abdominal or femoral surgery in the past year and 69 with a musculoskeletal disorder. The final sample covered 1439 participants, 1002 (69.6%) and 437 (30.4%) of whom were assigned to the development and validity datasets, respectively. Totally 188 (13.1%) were confirmed with NLBP, with 130 (13.0%) in the development dataset and 58 (13.3%) in the validation dataset. The participants aged 34.41 ± 9.34 years on average; their body mass index (BMI) was 21.92 ± 3.03, and 717 (50.0%) of them were female. More detailed baseline characteristics of the eligible participants for both the development and validation datasets are shown in Table 1.

**Table 1:** Baseline characteristics of eligible participants in the development and validation datasets

Variable	Whole dataset(*N* = 1439)	Development dataset(*N* = 1002)	Validation dataset(*N* = 437)
Individual characteristics			
Age	34.41 ± 9.34	34.16 ± 9.21	34.97 ± 9.64
BMI	21.92 ± 3.03	21.98 ± 3.07	21.80 ± 2.94
Average exercise times weekly	2.35 ± 1.25	2.37 ± 1.28	2.30 ± 1.16
Gender			
Female	722 (50.2)	518 (51.7)	204 (46.7)
Male	717 (49.8)	484 (48.3)	233 (53.3)
Educational level			
Middle school or below	228 (15.8)	163 (16.3)	65 (14.9)
High school	337 (23.4)	233 (23.3)	104 (23.8)
College degree or above	874 (60.7)	606 (60.5)	268 (61.3)
Marital status			
Singlehood	417 (29.0)	297 (29.6)	120 (27.5)
Married	1001 (69.6)	690 (68.9)	311 (71.2)
Divorced et al.	21 (1.5)	15 (1.5)	6 (1.4)
Smoking habits			
Non-smoking	1104 (76.7)	755 (75.3)	349 (79.9)
Smoking	259 (18.0)	195 (19.5)	64 (14.6)
No longer smoking	76 (5.3)	52 (5.2)	24 (5.5)
Drinking habits			
None	1032 (71.7)	710 (70.9)	322 (73.7)
A little(≤one bottle beer daily)	346 (24.0)	246 (24.6)	100 (22.9)
Much(>one bottle beer daily)	61 (4.2)	46 (4.6)	15 (3.4)
Place of residence			
City	334 (23.2)	231 (23.1)	103 (23.6)
Villages and towns	717 (49.8)	494 (49.3)	223 (51.0)
Countryside	388 (27.0)	277 (27.6)	111 (25.4)
Monthly income (CNY)			
< 1000	134 (9.3)	100 (10.0)	34 (7.8)
1000 ≤ < 5000	1006 (69.9)	702 (70.1)	304 (69.6)
5000 ≤ < 10,000	258 (17.9)	176 (17.6)	82 (18.8)
≥ 10,000	41 (2.8)	24 (2.4)	17 (3.9)
Work-related characteristics			
Main nature of work			
Brain	460 (32.0)	328 (32.7)	132 (30.2)
Brain & manual	739 (51.4)	506 (50.5)	233 (53.3)
Manual	240 (16.7)	168 (16.8)	72 (16.5)
The intensity of daily work			
Almost not	321 (22.3)	219 (21.9)	102 (23.3)
Light	765 (53.2)	537 (53.6)	228 (52.2)
Intergrade	310 (21.5)	219 (21.9)	91 (20.8)
Heavy	43 (3.0)	27 (2.7)	16 (3.7)
Vibration			
Systemic	43 (3.0)	31 (3.1)	12 (2.7)
Partial	292 (20.3)	199 (19.9)	93 (21.3)
None	1104 (76.7)	772 (77.0)	332 (76.0)
Psychosocial characteristics			
SF1	3.24 ± 0.64	3.23 ± 0.65	3.28 ± 0.61
SF2	3.19 ± 0.76	3.18 ± 0.76	3.22 ± 0.75
SF_PHYS	15.24 ± 2.00	15.20 ± 2.03	15.31 ± 1.94
SF_PSYCH	13.85 ± 2.21	13.77 ± 2.26	14.03 ± 2.09
SF_SOCIL	14.21 ± 2.34	14.24 ± 2.35	14.15 ± 2.31
SF_ENVIR	12.46 ± 2.20	12.45 ± 2.22	12.47 ± 2.14

Age, years; *BMI* Body mass index; Vibration means whether or not contacting vibration sources at work. Systemic includes feet or hip contact, and partial means hands contact; *SF1* Overall evaluation about quality of life, *SF2* Overall evaluation about health condition, *SF_PHYS* Physiology health domain, *SF_PSYCH* Psychological health domain, *SF_SOCIL* Social relation health domain, *SF_ENVIR* Environmental health domain

### NLBP risk model

The demographic and work-related factors significantly associated with NLBP in the basic model were as follows: age, BMI, average exercise times weekly, gender, educational level, the intensity of daily work, place of residence and monthly income (Table 2). The AUC of the basic model was 0.838 (95%*CI*: 0.798–0.878). Two psychosocial factors (the overall evaluation of health condition and physiological health) were added to the final risk model. The risk model had an excellent discriminating power with an AUC of 0.868 (95%*CI*: 0.830–0.905) and was significantly more effective than the basic model (0.868 vs 0.838, *P* < 0.001). Neither co-linearity nor interaction effects were significant.

**Table 2:** Univariate and multivariate logistic regression analysis of NLBP (Development dataset, *N* = 1002)

Variable	Univariate Analysis		Basic model		Risk model		
	*OR*(95%*CI*)	*P*	*OR*(95%*CI*)	*P*	Coefficient	*OR*(95%*CI*)	*P*
Age (years)	1.08 (1.06–1.11)	< 0.001	1.10 (1.07–1.13)	< 0.001	0.096	1.10 (1.07–1.13)	< 0.001
BMI (kg/m^2^)	1.18 (1.11–1.24)	< 0.001	1.16 (1.09–1.25)	< 0.001	0.143	1.15 (1.07–1.24)	< 0.001
Average exercise times weekly	0.82 (0.70–0.97)	0.019	0.69 (0.57–0.83)	< 0.001	−0.280	0.76 (0.62–0.92)	0.006
Female	1.39 (0.96–2.01)	0.084	1.75 (1.08–2.83)	0.024	0.584	1.79 (1.07–2.99)	0.026
Educational level		< 0.001		< 0.001			< 0.001
Middle school or below	1.00		1.00		0.000	1.00	
High school	0.99 (0.46–2.13)	0.980	2.80 (1.16–6.74)	0.022	0.820	2.27 (0.90–5.72)	0.082
College degree or above	2.52 (1.35–4.70)	0.004	8.11 (3.73–17.60)	< 0.001	1.906	6.72 (2.97–15.21)	< 0.001
Marital status		< 0.001					
Singlehood	1.00						
Married	5.09 (2.70–9.61)	< 0.001					
Divorced et al.	17.33 (5.24–57.30)	< 0.001					
The intensity of daily work		< 0.001		< 0.001			< 0.001
Almost not	1.00		1.00		0.000	1.00	
Light	3.18 (1.61–6.30)	0.001	2.59 (1.24–5.42)	0.012	0.723	2.06 (0.96–4.45)	0.065
Intergrade	5.11 (2.49–10.46)	< 0.001	5.50 (2.47–12.24)	< 0.001	1.342	3.83 (1.68–8.72)	0.001
Heavy	5.97 (1.97–18.07)	0.002	22.30 (5.87–84.62)	< 0.001	2.633	13.92 (3.36–57.72)	< 0.001
Place of residence		< 0.001		0.003			0.035
City	1.00		1.00		0.000	1.00	
Villages and towns	3.34 (1.82–6.13)	< 0.001	3.12 (1.53–6.38)	0.002	0.870	2.39 (1.14–5.00)	0.021
Countryside	2.43 (1.25–4.70)	0.009	1.83 (0.82–4.11)	0.140	0.438	1.55 (0.67–3.59)	0.307
Monthly income (CNY)		0.016		0.032			0.009
< 1000	1.00		1.00		0.000	1.00	
1000 ≤ < 5000	5.25 (1.63–16.88)	0.005	2.69 (0.77–9.37)	0.119	1.145	3.14 (0.85–11.58)	0.085
5000 ≤ < 10,000	4.86 (1.42–16.62)	0.012	1.82 (0.45–7.34)	0.400	0.901	2.46 (0.56–10.82)	0.233
≥10,000	10.78 (2.47–47.08)	0.002	8.46 (1.62–44.33)	0.011	2.744	15.55 (2.71–89.08)	0.002
Main nature of work		0.036					
Brain	1.00						
Brain & manual	1.20 (0.80–1.81)	0.370					
Manual	0.52 (0.27–1.02)	0.059					
Vibration		0.013					
Systemic	1.00						
Partial	0.36 (0.12–1.10)	0.074					
None	0.88 (0.33–2.35)	0.802					
Smoking habits		0.397					
Non-smoking	1.00						
Smoking	1.22 (0.80–1.86)	0.350					
No longer smoking	2.37 (0.47–11.93)	0.294					
Drinking habits		0.881					
None	1.00						
A little(≤one bottle beer daily)	0.95 (0.61–1.46)	0.798					
Much(>one bottle beer daily)	0.80 (0.31–2.07)	0.645					
SF1	0.45 (0.34–0.61)	< 0.001					
SF2	0.32 (0.24–0.42)	< 0.001			−0.741	0.48 (0.33–0.69)	< 0.001
SF_PHYS	0.68 (0.61–0.75)	< 0.001			−0.258	0.77 (0.68–0.88)	< 0.001
SF_PSYCH	0.78 (0.72–0.85)	< 0.001					
SF_SOCIL	0.88 (0.81–0.95)	0.001					
SF_ENVIR	0.86 (0.79–0.93)	< 0.001					

*OR* Odds ratio, *CI* Confidence interval, *BMI* Body mass index, Vibration means whether or not contacting vibration sources at work. Systemic includes feet or hip contact, and partial means hands contact; *SF1* Overall evaluation about quality of life, *SF2* Overall evaluation about health condition, *SF_PHYS* Physiology health domain, *SF_PSYCH* Psychological health domain, *SF_SOCIL* Social relation health domain, *SF_ENVIR* Environmental health domain, Basic model includes demographic and work-related factors; Risk model, basic model+SF2+ SF_PHYS

### NLBP pre-scoring system and risk category

The associated factors and corresponding scores for calculating the risk score of NLBP are presented in Table 3. The estimated probability, according to the proposed risk score, was expressed as:

1 $$ P\left(\mathrm{NLBP}\right)=\frac{1}{1+{e}^{-\left(-11.194+0.48\ast \left( total\ scores\right)\right)}} $$

**Table 3:** Scores of the associated factors in the pre-scoring system

Associated factor	Category	Score	AUC for single factor
Age (years)			0.698 (0.651–0.746)
	20 ≤ < 30	0	
	30 ≤ < 40	2	
	40 ≤ < 50	4	
	50 ≤ < 60	6	
BMI (kg/m^2^)			0.645 (0.594–0.696)
	< 18.5	0	
	18.5 ≤ < 24.0	1	
	24.0 ≤ < 28.0	2	
	≥28.0	3	
Average exercise times weekly			0.581 (0.531–0.632)
	0 ≤ ≤1	2	
	2 ≤ ≤3	1	
	≥4	0	
Gender			0.541 (0.488–0.594)
	Male	0	
	female	1	
Educational level			0.599 (0.550–0.648)
	middle school or below	0	
	high school	2	
	college degree or above	4	
The intensity of daily work			0.621 (0.572–0.670)
	almost not	0	
	light	2	
	intergrade	3	
	heavy	5	
Place of residence			0.599 (0.550–0.647)
	city	0	
	villages and towns	2	
	countryside	1	
Monthly income (CNY)			0.555 (0.504–0.605)
	< 1000	0	
	1000 ≤ < 5000	2	
	5000 ≤ < 10,000	4	
	≥10,000	6	
SF2			0.722 (0.675–0.769)
	1 ≤ ≤2	5	
	3	2	
	4 ≤ ≤5	0	
SF_PHYS			0.682 (0.635–0.729)
	5 ≤ < 11	4	
	11 ≤ < 14	3	
	14 ≤ < 17	2	
	17 ≤ ≤20	0	

The total score ranges from 0 to 38

*AUC* Area under the curve, *BMI* Body mass index, *SF2* Overall evaluation about health condition, *SF_PHYS* Physiology health domain

where −11.194 and 0.48 were the intercept and slope coefficient from the model, respectively. The risk of NLBP was calculated based on the total score, ranging from 0 to 38, with corresponding predicted probabilities from 0.0 to 99.9% (Table 4). The bootstrap-corrected AUC of the pre-scoring system was 0.861 (95%*CI*: 0.822–0.898).

**Table 4:** The total score and corresponding risk probability of NLBP

Total score	Risk probability (%)	Total score	Risk probability (%)
< 14	< 1.0	23	46.2
14	1.1	24	58.1
15	1.8	25	69.1
16	2.9	26	78.3
17	4.6	27	85.4
18	7.2	28	90.4
19	11.2	29	93.9
20	16.9	30	96.1
21	24.7	> 30	> 97.6
22	34.7		

**Fig. 2:**
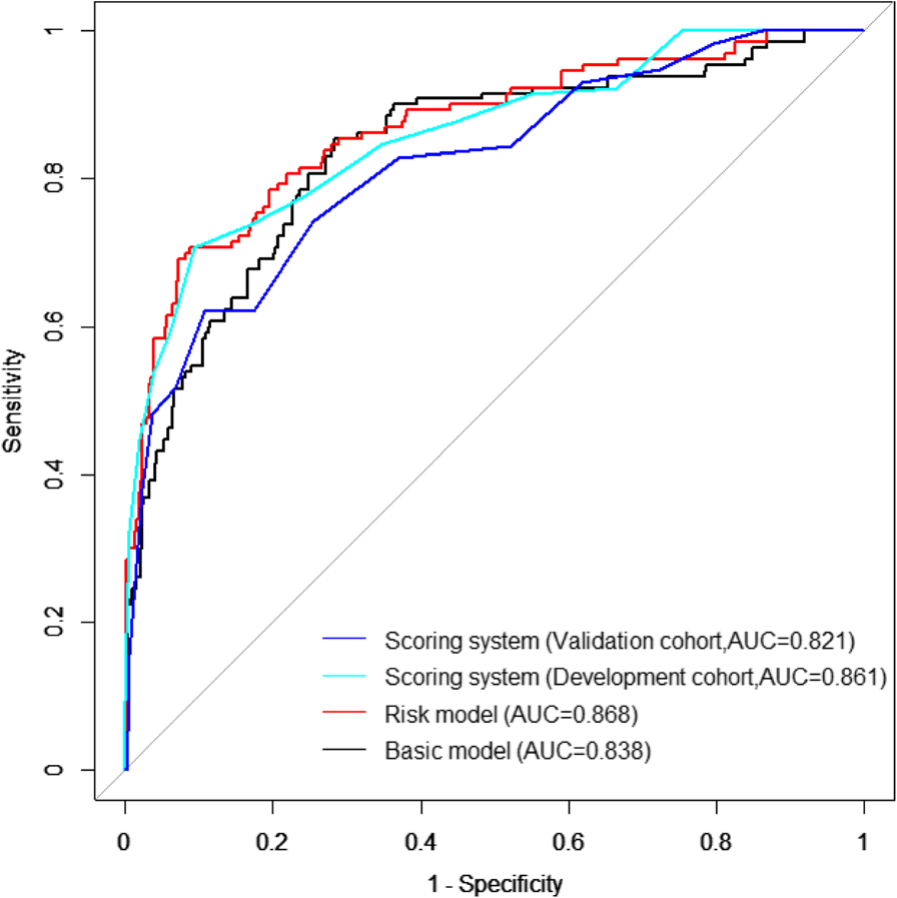
Receiver operator characteristic curve showing area under the curve for NLBP in general population (Notes: The basic model includes demographical and work-related factors. Risk model means basic model+SF2+ SF_PHYS. Scoring system means the sum of each factor score)

**Fig. 3:**
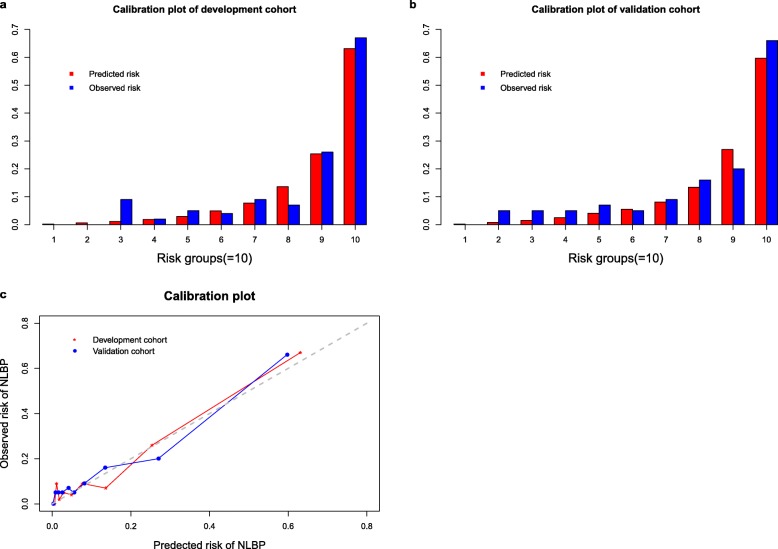
Calibration plot about observed versus estimated risk for NLBP in the development dataset and validation dataset respectively. **a** Calibration plot (Histogram) about observed risk versus estimated risk for NLBP in the development dataset with risk divided into ten groups by default. **b** Calibration plot (Histogram) about observed risk versus estimated risk for NLBP in the validation dataset with risk divided into ten groups by default. **c** Calibration plot (Line graph) about observed risk versus estimated risk for NLBP in the development dataset and validation dataset. The points with different colors indicate the observed risk against estimated risk

In the validation dataset, the AUC of the system was 0.821 (95%*CI*: 0.758–0.883). The receiver operating characteristics curves (ROCs) of the basic model, the risk model, and the system are shown in Fig. 2. The calibration plots of both datasets appeared no apparent over- or under-estimation (Fig. 3).

To illustrate the application of the risk score, consider a 30-year-old man with a height of 1.75 m, weight of 70.0 kg, average exercise times weekly of 2, education level of college degree or above, the intensity of daily work of intergrade, place of residence of urban, monthly income of > 10,000 RMB, overall evaluation about health condition of satisfied (SF2, 4 score), and physiology health of 14 score (ranging from 5 to 20 score). His total risk score is:

0 (Gender) + 2 (Age) + 1 (BMI) + 2 (Average exercise times weekly) + 4 (Educational level) + 3 (the intensity of daily work) + 0 (Place of residence) + 6 (Monthly income) + 0 (SF2) + 2 (SF_PHYS) = 20 from Table 3, and the estimated predicted probability that he has NLBP is 16.88% according to Formula 1. We also provided a convenient Excel tool for individuals to acquire the underlying risk of NLBP by entering their personal information (Additional file 1: Excel S1).

We created three NLBP risk categories: low risk (0 to 18 scores), intermediate risk (19 to 22 scores), and high risk (23 to 38 scores), to enhance the practical utility of the system. The categories were created by identifying the groups of scores that resulted in “significant” (*p*-value < 0.001) differences in the prevalence rate of NLBP between pairwise categories. The possibilities of developing NLBP in three categories in the development dataset were 4.3, 14.8, and 67.3%, respectively. The corresponding results in the validation dataset were 5.0, 15.5, and 66.7%, respectively (Table 5).

**Table 5:** Risk grades of NLBP

Risk category	Development Dataset, *N* = 1002			Validation Dataset, *N* = 437		
	Participants (n)	Incidence, n (%)	Odds Ratio (95% *CI*)	Participants (n)	Incidence, n (%)	Odds Ratio (95% *CI*)
Low-risk (0–18 score)	688	29 (4.3)	1.0	298	15 (5.0)	1.0
Intermediate-risk (19–22 score)	210	31 (14.8)	3.9 (2.3–6.7)	97	15 (15.5)	3.5 (1.6–7.4)
High-risk (23–38 score)	104	70 (67.3)	46.8 (26.9–81.4)	42	28 (66.7)	37.7 (16.5–86.1)

## Discussion

### Main finding

The prevalence rate of NLBP in our study was 13.1%, which is consistent with the findings of 10.0–49.0% in literature according to the definition of NLBP [5, 11–13, 36]. The validated pre-scoring system we developed based on ten factors had a high discriminative power at the bootstrap-corrected AUC of 0.861 and was strongly supported by the external validation (AUC of 0.821). These factors were age, BMI, average exercise times weekly, gender, educational level, the intensity of daily work, place of residence, monthly income, overall evaluation of health condition and physiology health. To our knowledge, this is the first simple and validated pre-scoring system for identifying the risk of NLBP among the general population. An excel evaluation tool was in the Additional file 1.

### Interpretation

Many cohort studies and meta-analyses have been conducted to explore the potential risk factors of NLBP, and the effects of some interventions have been confirmed by well-designed randomised control trials [37]. However, few of them focus on the readily available psychosocial data. In this research, we have not only studied demographic and work-related characteristics, but also concentrate on the psychosocial features. The results show that the overall evaluation of health condition (*OR* = 0.48) and physiology health (*OR* = 0.77) are two unneglectable factors. It indicates that the risk of NLBP may be reduced by alleviating the stress and tension and keeping optimistic.

Contrary to the results from the majority of literature, our findings show that the higher the income, the higher the risk of NLBP and so it is with the educational level. In our consideration, it may depict a picture in China context where the better-educated cohort is more likely to have access to a higher-income and more prone to being sedentary in the office. The realistic situation is that the cohort of higher-income tends to drive to work or travel, which keeps the waist muscles consistently in a state of intense stress. Yue et al. [38] pointed out that prolonged sitting and static posture are two potential risk factors of NLBP in China. Cocker et al. [39] and Hadgraft et al. [40] found that more occupational sitting is associated with higher income and education level in Australia. Other literature also gave a similar conclusion about the relation between NLBP and education in our findings [38, 41–46]. On the other side, the cohort of lower income cannot afford a car and therefore tends to go out on a walk or by bicycle. Although they are more likely to be exposed to vibration while working, the influence may be undermined by those mentioned above two significant factors. As it is seen, in the meantime, both the cohort of higher income and higher education, are consistent in doing physical exercises all through the communities in the developed world.

Some researchers have used different statistical models to predict the development of NLBP [23–25, 30], such as Classification Tree Model, Artificial Neural Network, Bayesian method, et al. Their outcomes are moderate and not validated by external population. The logistic regression model owns pretty well stability and easy-to-understand results, especially in medical research, so we use it in our study. A few researchers in their highly cited papers use different weighted methods based on regression coefficients to develop risk scores [47, 48]. In our research, we chose Sullivan’s approach, a generally accepted score construction method, to develop the scoring system, which will make it more stable [34].

### Clinical and decision-making implication

The present study derived and validated a pre-scoring system rather than a decision rule. It is to provide information that allows clinicians and patients to understand risks faced by patients, and then take actions to reduce the risk of NLBP. The factors incorporated in the constructed pre-scoring system are readily accessible data in general clinical work. The proposed system may help clinicians to identify patients at low risk of NLBP quickly. And among patients with a high risk of NLBP, a more detailed assessment of pain and a diagnostic test would then be needed to quantify the risk of NLBP adequately. Therefore, stratification of the risk level of NLBP patients according to the pre-scoring system is clinically relevant, particularly in disease prevention or care setting where efficient assessment is essential.

### Comparison with other risk score

Janwantanakul et al. [23] constructed a screening tool based on the previous history of LBP and psychological demand (assessed by the Job Content Questionnaire) with a good AUC of 0.76 (95% *CI* 0.68–0.83). Jensen et al. [28] used baseline clinical and psychosocial risk factors to predict patients with low, intermediate and high risk for an unsuccessful return to work, both initially and at 1-year, and yielded an excellent predictive effect. However, our pre-scoring system was developed among the general population whose population characteristic and risk exposure were different from those mentioned above. The AUC of the proposed system reached 0.821 in the validation dataset, demonstrating its excellent discrimination in the general population.

### Strengths and limitations

In this manuscript, our system enjoys the following properties: (1) It included a relatively large number of participants; (2) EM imputation was applied to utilize information as much as possible and ensuringd the robustness of the results; (3) the pre-scoring system was of high discriminating power as well as high stability.

Our study has several limitations as follows. First, it is not well suited to establish the causal relationship between exposure factors and outcome with a cross-sectional study. Therefore, the system in this study should be taken as a preliminary result. Secondly, there might be a bias if the findings are applied outside Guangzhou. Thirdly, we overlooked some potential risk factors in rehabilitation medicine or physical therapy (such as muscle imbalance) that may introduce some bias in our result. While some studies indicated that exercise could reduce muscle imbalance [49, 50], which stated that exercise, an essential variable in our model, is likely to explain the partial effects of muscle imbalance on NLBP. Finally, the bootstrap procedure, based on the developed risk score and did not include the variable selection step, might lead to the estimated bias of over-optimism in a way.

## Conclusions

We validated a pre-scoring system based on eight demographic and work-related features and two psychosocial factors that may be useful for assessing the risk of NLBP among the general population in Guangzhou.

## Additional file

Additional file 1: **Excel S1** A Scoring System for Nonspecific LBP among the general Population in Guangzhou. (XLSX 25 kb)

## Data Availability

The datasets used and/or analyzed during the current study are available from the corresponding author on reasonable request.

## Abbreviations

AUC: Area under the curve
BMI: Body mass index
EM: Expectation-maximum
EPV: Events per variable
IDI: Integrated discrimination improvement
LBP: Low back pain
NLBP: Nonspecific low back pain
NRI: Net reclassification improvement
ROC: Receiver operating characteristics curve
TRIPOD: Transparent reporting of a multivariable prediction model for individual prognosis or diagnosis
